# Investigating Chaperonin-Containing TCP-1 subunit 2 as an essential component of the chaperonin complex for tumorigenesis

**DOI:** 10.1038/s41598-020-57602-w

**Published:** 2020-01-21

**Authors:** Anne E. Showalter, Ana C. Martini, Daniel Nierenberg, Kristen Hosang, Naima Ahmed Fahmi, Priya Gopalan, Amr S. Khaled, Wei Zhang, Annette R. Khaled

**Affiliations:** 1 Division of Cancer Research, Burnett School of Biomedical Science, College of Medicine, University of Central Florida, Orlando, FL 32827 USA; 2 Department of Computer Science, University of Central Florida, Orlando, FL 32827 USA; 3 Genomics and Bioinformatics Cluster, University of Central Florida, Orlando, FL 32816 USA; 4 Oncology, Department of Internal Medicine, Orlando VA Medical Center, Orlando, FL 32827 USA; 5 Pathology and Laboratory Medicine, Orlando VA Medical Center, Orlando, FL 32827 USA

**Keywords:** Breast cancer, Cell growth

## Abstract

Chaperonin-containing TCP-1 (CCT or TRiC) is a multi-subunit complex that folds many of the proteins essential for cancer development. CCT is expressed in diverse cancers and could be an ideal therapeutic target if not for the fact that the complex is encoded by eight distinct genes, complicating the development of inhibitors. Few definitive studies addressed the role of specific subunits in promoting the chaperonin’s function in cancer. To this end, we investigated the activity of CCT2 (CCTβ) by overexpressing or depleting the subunit in breast epithelial and breast cancer cells. We found that increasing total CCT2 in cells by 1.3-1.8-fold using a lentiviral system, also caused CCT3, CCT4, and CCT5 levels to increase. Likewise, silencing *cct2* gene expression by ~50% caused other CCT subunits to decrease. Cells expressing CCT2 were more invasive and had a higher proliferative index. CCT2 depletion in a syngeneic murine model of triple negative breast cancer (TNBC) prevented tumor growth. These results indicate that the CCT2 subunit is integral to the activity of the chaperonin and is needed for tumorigenesis. Hence CCT2 could be a viable target for therapeutic development in breast and other cancers.

## Introduction

The hallmarks of cancer (uncontrolled proliferation, genomic instability, metastasis, etc.) reveal the complex nature of this disease and the challenges faced developing effective therapeutics^1,2^. Cancer does, however, have an “Achilles heel” and that is its dependency or addiction on major cellular events or processes like transcription, translation, splicing, protein degradation and protein-folding^3^. In healthy cells, such conserved and essential processes are rigorously regulated by the proteostasis network (PN) to ensure proteome balance. In order to maintain proteome integrity, the cellular proteome must be synthesized, folded into its native structure, and, when no longer needed, degraded and the amino acids recycled^4,5^. Chaperones and chaperonins are key players in the PN^6^. Unlike healthy, non-transformed cells, the PN of cancer cells is taxed to produce proteins involved in survival, angiogenesis, migration, proliferation which are essential for tumor formation, progression and metastasis. Cancer cells have a higher dependency on molecular chaperones and are uniquely challenged due to imbalances caused by chromosomal abnormalities and overexpression of oncogenes, ultimately leading to cellular stress^7^. As example, inhibitors of Heat Shock Protein 90 (HSP90) showed promising outcomes in the treatment of metastatic breast cancer^8^. However, despite being in clinical trials since 1998, the success of HSP90 inhibitors in clinical trials remains mixed^9–11^. Reasons such as dose-limiting toxicity, incomplete inhibition of HSP90, and insufficient downregulation of client proteins impeded the clinical use of current HSP90 inhibitors^12,13^. In recent years, another class of protein-folding complexes, called chaperonins, gained interest with the recognition that cancer cells highly express these mediators of protein-folding^14–23^. The cytosolic, eukaryotic chaperonin-containing TCP-1 (CCT) is an emerging player in neoplastic transformation with potential for development as a novel diagnostic marker and therapeutic target.

CCT is a type II chaperonin composed of two stacked rings, back-to-back, consisting of eight paralogous subunits (CCT1-8 or CCTα, β, γ, δ, ε, ζ, η, θ)^24^. The two rings form a central cavity in which newly synthesized or misfolded proteins can be sequestered and folded in a ATP dependent manner^25,26^. Previous reports estimated that in eukaryotic cells, CCT facilitates the folding of approximately 10% of newly synthesized proteins, co- and post-translationally^27–29^, with actin and tubulin being obligate substrates^30^. In cancer cells, CCT could potentially fold more proteins, with substrates including oncogenic proteins and mediators such as KRAS, STAT3, among others^14,20,31–33^. Genomic amplification of CCT or upregulation of gene expression suggests that CCT could be a marker for oncogenesis^34^. Previous studies reported that the expression levels of different CCT subunits are upregulated in various cancers, such as CCT8 and CCT3 in hepatocellular carcinoma^18,22^ or glioblastoma^35^, and CCT2 in prostate, breast, and lung cancers^14,15,21^. Our lab discovered a CCT inhibitor called CT20p that kills cancer cells in a CCT-dependent manner. Cancer cells in which CCT was inhibited were resistant to CT20p killing, while cells in which CCT was increased were susceptible^14,16^. However, the complexity of CCT, with its multiple subunits, and the lack of a full understanding of CCT substrate selectivity *in vivo*, are some of the challenge that hinder the development of promising therapeutics like CT20p.

The goal of this study is to determine whether the contribution of CCT chaperonin complex to carcinogenesis can be elucidated by focusing on a single subunit, CCT2. We and others showed that CCT2 is increased in breast, prostate, and lung cancer as compared to normal tissues and that CT20p directly interacts with CCT2^14–16,21^. But whether CCT2 is an essential component of the chaperonin’s activity in cancer cells and promotes tumorigenesis is unknown. To address this, we expressed CCT2 as well as silenced *cct2* gene expression in epithelial and breast cancer cells. Our study revealed that the levels of CCT2 influence other CCT subunits and that its expression drives invasiveness and proliferation. CCT2 was needed for tumor growth, indicating that this single subunit could be a viable therapeutic and diagnostic target in cancer.

## Results

### CCT is highly expressed in breast cancer and inversely correlates with patient survival

**Figure 1:**
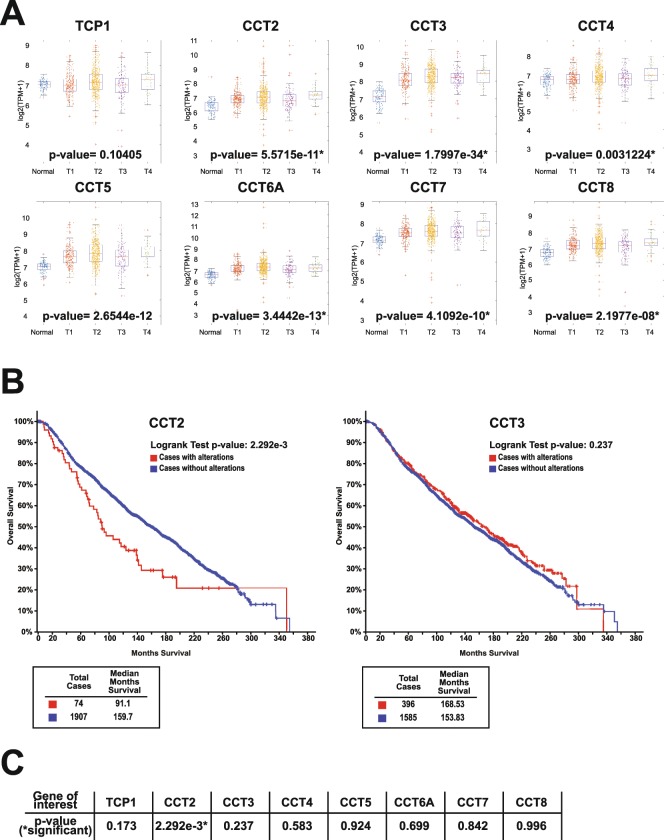
Expression levels of CCT subunits increase with breast cancer stage, while CCT2 levels inversely correlate with patient survival. Expression levels of all eight CCT subunits were analyzed according to stage and survival in breast cancer patients. (**A**) Plots are shown comparing expression levels of all eight CCT subunits in normal (n = 116) and breast cancer tissues (n = 1110) across stage as indicated by T1-T4. The p-value shown in the box was calculated based on determining the Pearson Correlation Coefficients between the CCT expression level and cancer stage. Source was the Xena Public Data Hubs. (**B**) Kaplan-Meier plots comparing survival in patients with and without alterations in CCT2 and CCT3 genes. (**C**) Table showing the p-values comparing patient survival with genomic alterations of all CCT subunits. Source was the TCGA database using the cBioPortal for cancer genomics^60,62^.

The CCT complex is encoded by eight different genes (*tcp1*, *cct2-8*). To determine whether select subunits were upregulated in breast cancer and whether this changed with cancer stage, we performed a comprehensive analysis of the mRNA-sequencing (RNA-seq) data in 1110 breast cancer tumor samples and 116 normal tissue samples from a database compiled by the Akbani lab^36^. As shown in Fig. 1A, when comparing breast cancer to normal tissue, we found statistically significant increases in the majority of the genes expressing CCT subunits. This increase in CCT expression was sustained through late stages of the disease. Our previous publication reported that CCT levels were independent of hormone receptor status^16^, suggesting that the chaperonin levels could be altered in triple negative breast cancer (TNBC). Additional investigation using the same database showed that expression levels for all CCT subunits were significantly higher in TNBC tumors compared to normal tissue (Fig. S1A). Breast cancer patient survival data obtained from the Kaplan-Meier Plotter (KMPlotter) dataset showed that patient survival inversely correlated with CCT levels in tumor tissue (Fig. S1B), suggesting that high expression of the chaperonin associates with poor patient prognosis. While these data indicate that CCT could be a good therapeutic target whose inhibition positively impacts patient outcomes, targeting eight genes for inhibition is a challenge difficult to surmount. It would be ideal to target a single CCT subunit by demonstrating its essential role in promoting the chaperonin’s activity. To this end, we found that CCT2 protein levels, but not CCT3 as example, varied among breast cancer cell lines, with highest levels of CCT2 in TNBC cells and lowest in luminal A types and non-transformed breast epithelial cells (Fig. S1C,D; CCT3 shown as a representative of other subunits). The importance of CCT2 in breast cancer was further examined with patient data from The Cancer Genome Atlas (TCGA), showing that only genomic alterations in CCT2 resulted in statistically significant differences in patient survival (Fig. 1B,C). Patients with genomic alterations in CCT2 (mainly gene amplification or increased mRNA) died almost 70 months sooner than patients without changes in CCT2. These findings led us to investigate the role of CCT2 in cancer progression using CCT2 overexpressed or depleted cells.

### CCT2 expression promotes invasiveness and increased levels of other CCT subunits

**Figure 2:**
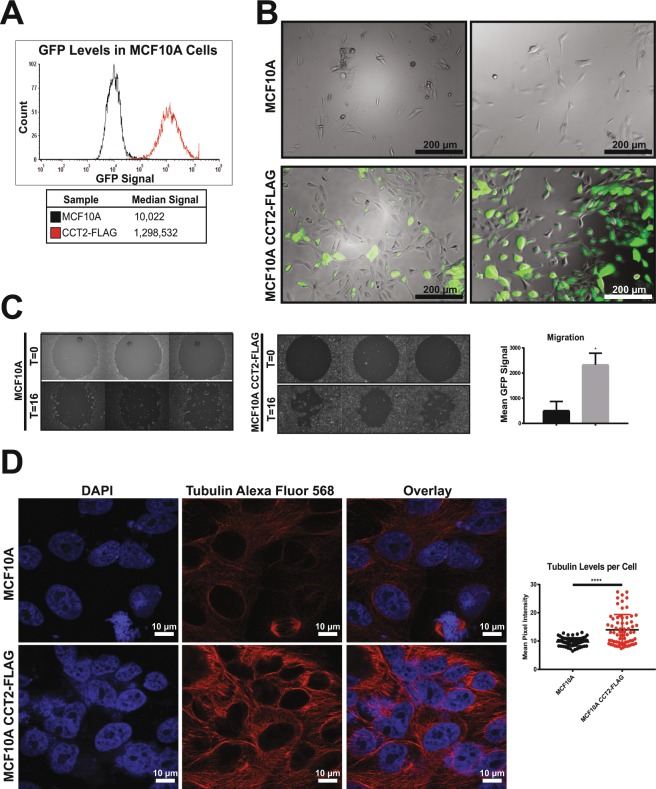
Expression of CCT2-FLAG causes phenotypic changes in MCF10A cells. MCF10A cells expressing CCT2-FLAG show (**A**) expression of plasmid-driven GFP measured by flow cytometry and (**B**) changes in morphology (100X). (**C**) Migration of MCF10A and MCF10A CCT2-FLAG cells was measured after 16 hours (T = 16) using the Oris Migration assay as described in Materials and Methods by detection of cells loaded with CFSE in the zone cleared by the plug. Start of the experiment (T = 0) is shown for comparison. Significance was determined using paired student’s T test with p-value = 0.0144. (**D**) MCF10A and MCF10A CCT2-FLAG cells were stained with DAPI (nucleus) and tubulin (Alexa Fluor 568) and assessed by confocal microscopy using Zeiss LSM 710 confocal microscope (100X). Tubulin levels were quantified per cell based on the mean pixel intensity. Significance was determined using paired student’s T test with p-value < 0.0001. Data shown is representative of three experiments.

**Figure 3:**
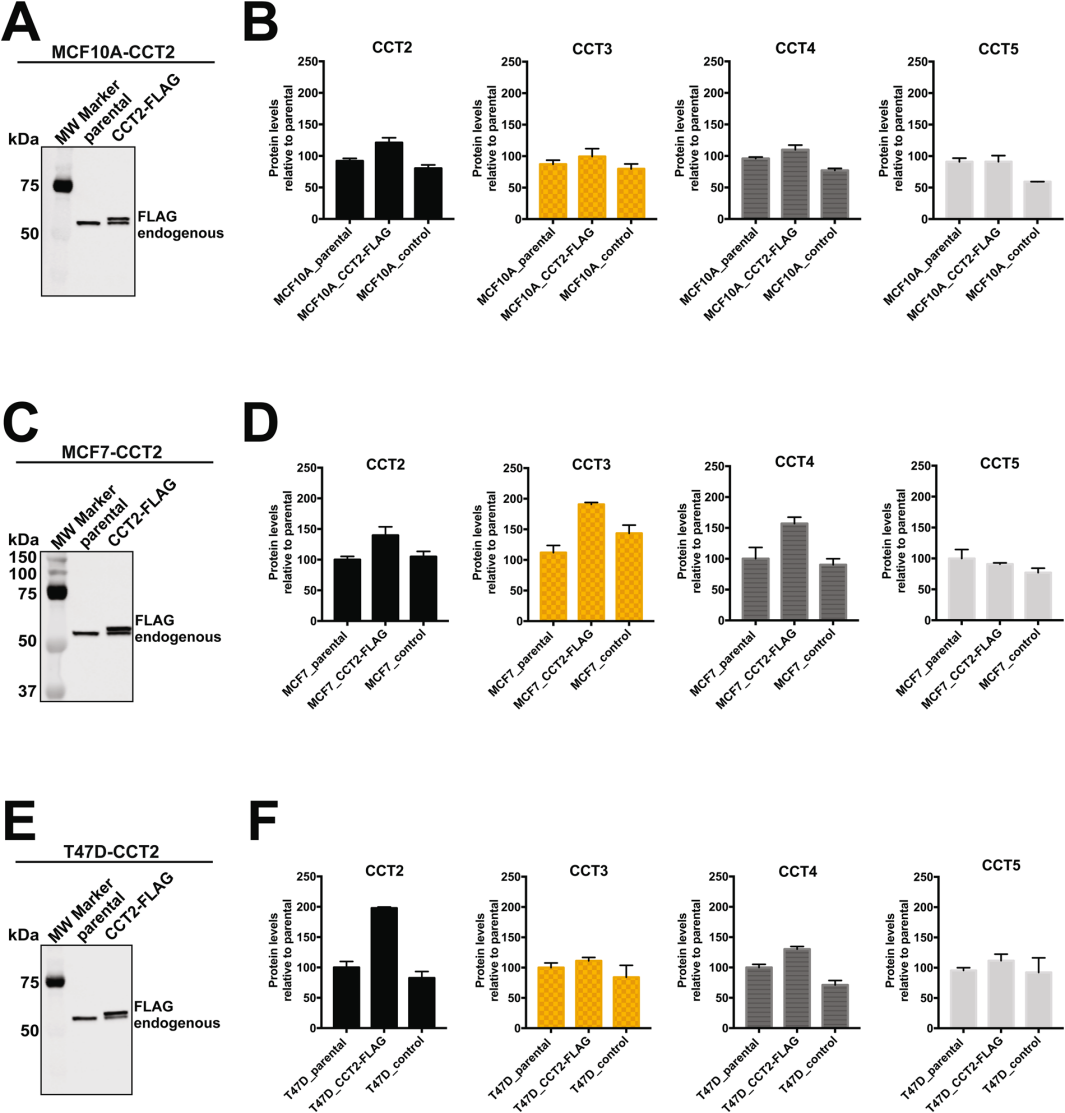
Expression of CCT2-FLAG leads to increased levels of other CCT subunits. Relative protein levels of CCT2, CCT3, CCT4 and CCT5 were determined by immunoblot analysis in cells stably expressing CCT2-FLAG. (**A**,**C**,**E**) Immunoblot of MCF10A (**A**), MCF7 (**C**) and T47D (**E**) to determine CCT2 levels in Parental (untransfected) and CCT2-FLAG cells. Note that CCT2-FLAG band runs at a slightly higher molecular weight than endogenous CCT2, confirming the presence of FLAG-tagged protein. The fold increase of total CCT2 levels (compared to Parental) in MCF10-CCT2, MCF7-CCT2 and T47D-CCT2 were 1.3, 1.5 and 1.8 respectively. Total CCT2 = endogenous + CCT2-FLAG. Cropped blots for CCT2 is shown; refer to Supplemental Fig. S3 for full blot and total protein images (used for signal normalization). (**B**,**D**,**F**) Relative protein levels between Parental, CCT2-FLAG and Control (GFP vector only) of CCT2, CCT3, CCT4 and CCT5 calculated from immunoblot analyses. The bar graphs represent protein levels relative to Parental cells. Signal was normalized to total protein as previously described^14^ and in Materials and Methods. Error bars represent the mean with s.e.m of technical replicates. Representative data of at least three independent experiments is shown. Refer to Supplemental Fig. S3 for full blot images.

In order to evaluate the effects of increased CCT2 in cells, we generated two versions of a plasmid, FLAG-CCT2 and CCT2-FLAG, with the FLAG tag on the N-terminal or C-terminal of CCT2 respectively (Fig. S2A) and used a third-generation lentiviral system for gene delivery. To determine transfection efficiency, the generated plasmids were designed to independently express GFP. K562 cells were used for evaluation of initial transduction efficiency, which was acceptable for both constructs as determined by flow cytometry (Fig. S2B). Immunoblots blots for CCT2 and FLAG were performed to confirm expression of FLAG-tagged CCT2 (Fig. S2C,D,G). We selected the CCT2-FLAG construct to continue our studies. To determine if the FLAG-tagged CCT2 was being incorporated in the CCT complex, we performed a pulldown experiment using anti-FLAG agarose beads. The immunoprecipitate was blotted for FLAG, CCT2, and CCT5, since CCT2 and CCT5 make direct contact in the complex^37^. The results obtained from the pulldown were suggestive that CCT2-FLAG associated with the chaperonin complex (Fig. S2E,F). Next, the immortalized breast epithelial cell line, MCF10A, was transduced with CCT2-FLAG and the GFP signal was used to measure transduction efficiency (Fig. 2A,B). Increased expression of CCT2 had pleiotropic effects on the cells. MCF10A-CCT2-FLAG cells were morphologically different than MCF10A cells, having a more cuboidal appearance and clumping during growth (Fig. 2B). Note that these are qualitative not quantitative assessments. Increased migration (Fig. 2C) and polymerized tubulin or microtubules (Fig. 2D) correlated with expression of CCT2-FLAG and are consistent with increased CCT complex activity, since actin and tubulin are obligate CCT client proteins^38–40^. We confirmed the presence of FLAG-tagged CCT2, as well as endogenous CCT2, by immunoblotting protein lysates from MCF10A-CCT2-FLAG cells for CCT2 (Fig. 3A). The total CCT2 levels (endogenous + CCT2-FLAG) increased ~1.3 fold. Interestingly, endogenous levels of CCT3, CCT4 and CCT5 also increased in MCF10A-CCT2-FLAG cells, especially in comparison to the cells expressing the lentiviral control plasmid (Fig. 3B).

To show that results from MCF10A cells were not cell specific, we generated two luminal A breast cancer cell lines, MCF7 and T47D, that stably expressed CCT2-FLAG. Total CCT2 protein levels (endogenous + CCT2-FLAG) were 1.5-1.8 times higher in these cells compared to controls (Fig. 3C,E). Levels of endogenous CCT3, CCT4 and CCT5 also variably increased compared to lentiviral controls (Fig. 3D,F). Blot images, replicates, and total protein are shown in Supplemental Data (Fig. S3A–G). We confirmed that the expressed CCT2-FLAG associated with other CCT subunits by immunoprecipitating CCT2-FLAG from lysates made from T47D-CCT2-FLAG cells with anti-FLAG agarose beads and blotting for CCT5 and CCT3 (Fig. S4A,B). To determine whether CCT2-FLAG was incorporated in the oligomeric complex or existed as a monomeric protein, native PAGE using T47D CCT2-FLAG cell lysates was performed. We found that most of the CCT2-FLAG was in the CCT oligomeric complex and a fraction existed as free monomer (~24% of the total FLAG signal). Endogenous CCT2 and CCT3 were also detected in the oligomeric complex along with CCT2-FLAG (Fig. S4C). While the total levels of CCT2 were increased (endogenous + CCT2-FLAG), we noted that the amounts of endogenous CCT2 were reduced in CCT2-FLAG-expressing cells (Fig. S4C). Whether this was due to a negative effect of monomeric or oligomeric CCT2-FLAG is yet to be determined. These results do suggest that CCT2-FLAG was influencing the levels of other CCT subunits, likely as part of the oligomeric complex, and while additional activities as a monomer cannot be ruled out, CCT2, is essential for the activity of the chaperonin complex and bears further investigation.

### Increased expression of CCT2 promotes cell proliferation

**Figure 4:**
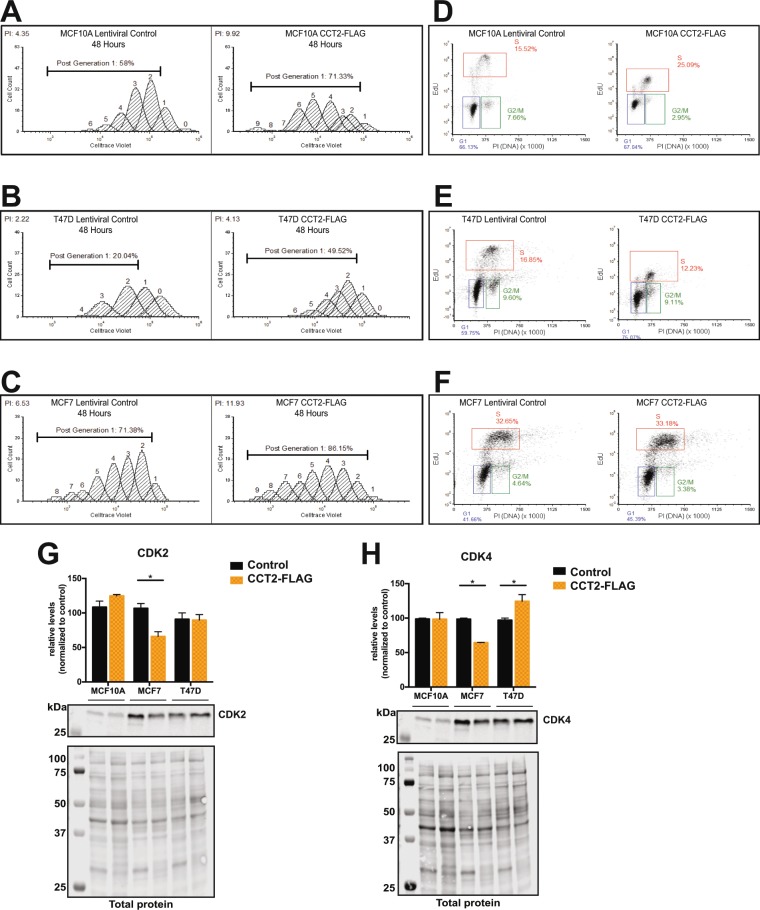
Expression of CCT2-FLAG promotes cell proliferation. MCF10A, T47D, and MCF-7, Lentiviral Control (Control) and CCT2-FLAG cells were analyzed for proliferation by flow cytometry and immunoblot. (**A**–**C**) Proliferation was assessed using Celltrace Violet Fluor® for 48 hours by flow cytometry (Cytoflex). Generation time and Proliferation Index (upper left) was calculated with FCS Express 6 software. The percent of original cells post-generation 1 is shown. Representative data of three experiments is shown. (**D**–**F**) Cell cycle phase was determined using Clickit EdU Alexa 647 and propidium iodide staining and analyzed by flow cytometry (Cytoflex) using FCS Express 6 software. Representative data of three experiments is shown. (**G**,**H**) Analyses of immunoblots is shown comparing CDK2 and CDK4 levels between control cells (black bars) and CCT2-FLAG (yellow bars) cells. The bar graphs represent protein levels relative to the control cells in each dataset. Signal was normalized to total protein as previously described^14^ and in Methods. (**G**) CDK2 level was significantly decreased in MCF7-CCT2 compared to MCF7-Control cells (*p = 0.0220). (**H**) CDK4 level was significantly decreased in MCF7-CCT2 compared to MCF7-Control cells (*p = 0.0161) and significantly higher in T47D-CCT2 (*p = 0.0426). Error bars represent the mean with s.e.m. Two-way ANOVA (Sidak’s multiple comparison test) was used to calculate significance between control and CCT2-FLAG signal. Blots were performed once with technical replicates for each lysate. Cropped blot for CDKs is shown. Refer to Supplemental Fig. S4C,D for full blot images.

Mediators of cell cycling, such as cyclin dependent kinases (CDKs) and cyclins, are potential CCT client proteins. We show this through a bioinformatics analysis of possible CCT interactors that are involved in cell cycling and whose expression co-occurs with CCT2 in breast cancer (Table 1). To determine whether expressing CCT2 could promote cell growth, we examined cell division using a dye dilution method. In our initial assessment of cell division, we observed that cells stably expressing the control lentiviral plasmid (no CCT2-FLAG) grew more slowly compared to non-transduced parental cells (Fig. S5A). This was shown by others and could be an effect attributed to the use of polybrene^41^. We realized that to detect a pro-growth effect mediated by CCT2 in the lentiviral transduced cells, we would need to evaluate cells after several passages to enable the protein-folding activity to rescue proliferation. This was observed as shown in Fig. 4. We found that MCF10A-CCT2-FLAG, MCF7-CCT2-FLAG, and T47D-CCT2-FLAG cells, loaded with CellTrace Violet for 48 hours, doubled their proliferative index and increased generation time as compared to lentiviral control cells similarly passaged (Fig. 4A–C). We further noticed minimal cell cycle arrest by assessing uptake of EdU (5-ethynyl-2 deoxyuridine), a nucleoside analogue, and PI (propidium iodide), a DNA-binding dye; although a reduction in EdU staining was noted in T47D-CCT2-FLAG and MCF10A-CCT2-FLAG cells, which could reflect growth pressure on these cells (Fig. 4D–F). For this reason, we examined proliferation of CCT2-FLAG-expressing and lentiviral control cells, loaded with CellTrace Violet, for 120 hours and found that proliferation of T47D-CCT2-FLAG (Fig. S5B) and MCF7-CCT2-FLAG (not shown) cells increased compared to lentiviral control cells, but MCF10A-CCT2-FLAG cells did not (Fig. S5B). However, this was likely due to MCF10A-CCT2-FLAG cells reaching conditions of confluency sooner than comparable numbers of lentiviral control cells. In support of CCT2 driving cell division, we examined the levels of CDK4 and CDK2, kinases that are involved in the G1-S phases of the cell cycle. These CDKs could be potential substrates or interactors of CCT (Table 1). CDK2 was slightly increased in the MCF10A-CCT2-FLAG cells (Fig. 4G), and CDK4 was significantly increased in T47D-CCT2-FLAG cells (Fig. 4H). Blot images and total protein are shown in Supplemental Data (Fig. S4D,E). Decreases in CDK2 and CDK4 protein levels in the MCF-7-CCT2-FLAG line indicated that other cell cycle proteins are likely promoting proliferation in these cells (examples in Table 1). We concluded that expressing the single CCT2 subunit induced cell proliferation likely through the activity of the chaperonin complex that increased key cell cycling proteins like CDKs and others.

**Table 1:** Cell cycle genes that correlate with CCT2 based on breast cancer stage.

Cell cycle genes	Co-occurrence with CCT2	Pearson CC stage I	Pearson CC stage III/IV
BUB1	Yes	None	0.449 (3.71E-11)
PRKDC	Yes	0.582 (4.30E-14)	0.441 (8.28E-11)
PLK1	Significant	None	0.435 (1.61E-10)
YWHAQ	Significant	0.665 (2.88E-19)	0.435 (1.65E-10)
PCNA	Significant	0.585 (3.32E-14)	0.43 (2.63E-10)
MCM2	Yes	None	0.424 (4.91E-10)
GSK3B	Significant	0.555 (1.04E-12)	0.412 (1.79E-09)
SMC3	Significant	0.636 (2.96E-17)	0.411 (1.87E-09)
STAG2	Significant	0.588 (2.17E-14)	0.4 (5.51E-09)
YWHAG	Yes	0.688 (5.76E-21)	0.506 (3.13E-14)
SMC1A	Significant	None	0.494 (1.63E-13)
CCNA2	Significant	None	0.484 (5.77E-13)
MCM4	Significant	0.559 (6.90E-13)	0.482 (6.94E-13)
SKP1	Significant	0.594 (1.03E-14)	0.48 (9.13E-13)
CDK2	Significant	0.7 (6.26E-22)	0.478 (1.15E-12)
CDK1	Significant	None	0.476 (1.55E-12)
BUB1B	Mutual Exclusivity	None	0.467 (4.57E-12)
BUB3	Yes	0.569 (2.27E-13)	0.456 (1.58E-11)
MDM2	Significant	None	0.604 (5.79E-21)
YWHAB	Significant	0.703 (3.72E-22)	0.583 (2.50E-19)
MAD2L1	Significant	None	0.553 (3.41E-17)
CDC23	Significant	0.703 (3.16E-22)	0.543 (1.56E-16)
YWHAZ	Significant	0.577 (8.10E-14)	0.535 (5.79E-16)
CDK4	Significant	0.695 (1.40E-21)	0.53 (1.01E-15)
CCNB1	Significant	None	0.52 (4.47E-15)
CDC27	Yes	None	0.516 (8.39E-15)
RAD21	Significant	0.579 (6.56E-14)	0.513 (1.31E-14)

### CCT2 loss leads to reduced viability and tumor growth

**Figure 5:**
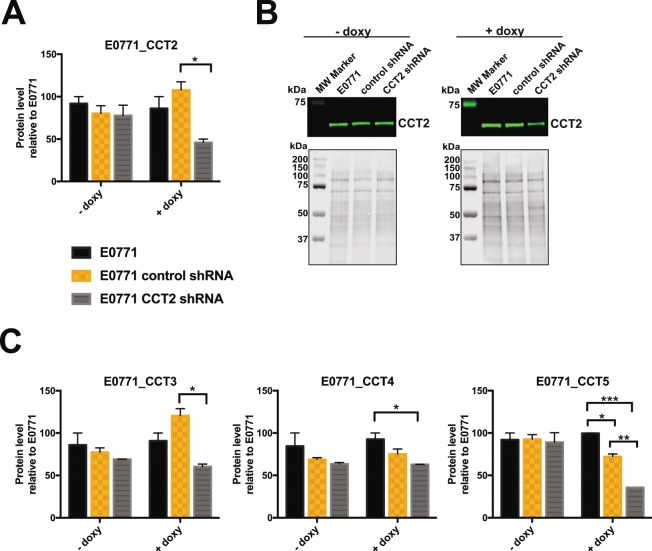
CCT2 depletion leads to the decreased levels of other CCT subunits. Using a doxycycline inducible lentiviral system to express CCT2 shRNA, CCT2 was knocked down in E0771 cells and relative levels of CCT2, CCT3, CCT4, and CCT5 in E0771, E0771 control shRNA, and E0771 CCT2 shRNA cells treated with (+doxy) or without (−doxy) 0.5 μg/ml doxycycline were determined by immunoblotting. (**A**) CCT2 levels decreased significantly (p = 0.0140) upon expression of CCT2 shRNA when compared to control shRNA in the doxy group. (**B**) Images of representative blots used for the analysis of CCT2 levels shown in A. Note the decrease in CCT2 signal in E0771-CCT2shRNA in the presence of doxy while CCT2 signal remains constant in the no doxy group. Total protein image corresponding to the blot above shows equal load between the lanes. Images were cropped to show CCT2 band only. Refer to Supplemental Fig. S7A,B for full blot images. (**C**) Relative levels of CCT3, CCT4, and CCT5 in E0771, E0771 control shRNA and E0771 CCT2 shRNA were also assessed from immunoblot data shown in Supplemental Fig. 7A–D. Two-way ANOVA analysis performed using Tukey’s multiple comparison test between -doxy and +doxy groups (p < 0.05). In + doxy group CCT3 *p = 0.0261. In +doxy group CCT4 *p = 0.0241, CCT5 *p = 0.0290 **p = 0.0078, ***p = 0.0004. Error bars represent the mean with s.e.m of technical replicates. Representative data of three independent experiments is shown.

Having shown the role of CCT2 in promoting growth and invasiveness of cells, we next examined the effects of CCT2 loss. We initially confirmed that CCT2 is essential for cells by silencing the *cct2* gene using a small interfering RNA (siRNA) approach targeting four different regions of the transcript (Fig. S6A). Since the introduction of CCT2 siRNA proved lethal to cells, a Tet-on shRNA inducible system was used to control CCT2 depletion and a targeting approach using a single shRNA was chosen. Using the TNBC cell line, MDA-MB-231, as the test model, we optimized the dose of doxycycline (doxy) and observed expression of the CCT2 shRNA after 72 hours as indicated by GFP co-expression (Fig. S6B). Outcomes were loss of viability, as evidenced by reduced adherence (Fig. S6C) and migration (Fig. S6D) in *cct2*-silenced MDA-MD-231 cells. Having shown that the inducible system was successful in the MDA-MB-231 cells, we transduced the E0771 murine TNBC cells to assess both *in vitro* and *in vivo* effects of CCT2 loss. Induction of *cct2*-gene silencing in E0771 cells with doxy caused an approximate 50% reduction of CCT2 protein (Fig. 5A,B). Reduction of total CCT2 led to concomitant decreases in CCT3, CCT4, and CCT5 (Figs. 5C, S7A–D). This data supports that CCT2 influences the levels of other subunits, as shown when overexpressing CCT2 (Fig. 3) or depleting CCT2 (Fig. 5).

**Figure 6:**
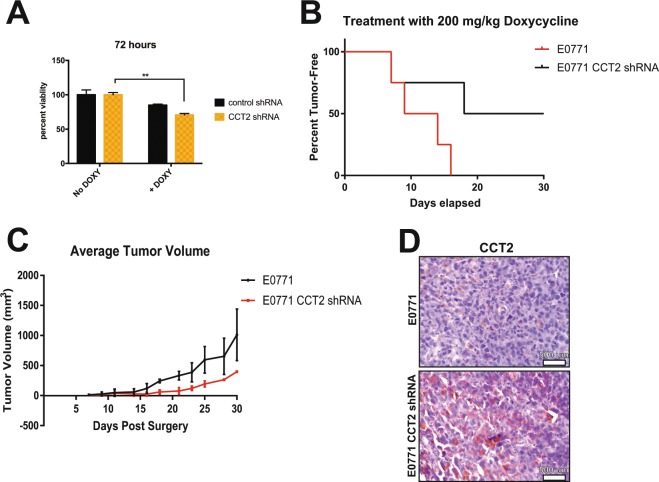
CCT2 depletion kills TNBC cells and inhibits tumor growth in syngeneic model of TNBC. (**A**) Viability of E0771 cells expressing control or CCT2 shRNA treated with or without 0.5 μg/ml doxycycline (doxy) was determined 96hrs after treatment using Live/Dead assay as described in Materials and Methods. Silencing *cct2 in vitro* decreased viability by ~25% in E0771 cells compared to control shRNA. B-C) E0771 and E0771 cells expressing the inducible CCT2 shRNA were orthotopically implanted in C57BL/6 mice (n = 4). Four days post-tumor implantation, mice were fed 200 mg/kg doxycycline chow. Tumor appearance (p-value = 0.0786 for survival curve) (**B**) and volume (**C**) were assessed by caliper measurements as described in Materials and Methods for 4 weeks. Data shown is representative of two experiments, each with n = 4 mice per group. (**D**) CCT2 protein levels in tumors from mice at experimental endpoints were assessed by immunohistochemistry (IHC). Representative data is shown.

We further demonstrated that *in vitro* induction of CCT2 shRNA caused the death of E0771 cells (Fig. 6A). Using the E0771/C57BL/6 syngeneic TNBC model, we assessed the *in vivo* effects of decreasing CCT2. E0771 (control and inducible CCT2 shRNA) cells were orthotopically implanted in the mammary fatpad of female C57BL/6 and, after 4 days, mice were fed chow containing doxy. We previously established that the doxy chow had no impact on tumor growth in mice (not shown). We found that 50% of the mice implanted with E0771-CCT2shRNA cells, receiving the doxy feed, developed tumors by 30 days and the remaining 50% of the mice did not develop tumors. In contrast, 100% of the control mice developed tumors by 16 days (Fig. 6B). This experiment was repeated with similar results. Note that the tumors that did grow in the *cct2-*silenced mice were smaller (Fig. 6C). While these results were consistent with findings of CCT2 knockdown efficiency using the inducible shRNA system (Fig. 5A,B), we determined whether CCT2 was actually depleted in the 50% of the mice implanted with E0771-CCT2shRNA that grew tumors. Tumor tissues were recovered from these mice for immunohistochemistry (IHC) analysis of CCT2. We found that these tumors had detectable levels of CCT2 that were comparable to control mice (Fig. 6D). We concluded that, in these mice, *cct2*-gene silencing was less efficient, enabling tumors to grow. These results confirm the data shown herein that CCT2 is an essential component of the chaperonin complex and supports the tumorigenic process.

## Discussion

Our focus on CCT2 as a potential therapeutic target for inhibition of chaperonin activity initiated upon discovering correlations between CCT2 expression and reduced breast cancer patient survival. Increased CCT2 expression was sustained through all breast cancer stages and correlated with poor prognosis in patients. Since the CCT complex is formed by eight different subunits, the importance of a single subunit, like CCT2, was undetermined. By overexpressing CCT2 in select breast epithelial and luminal A breast cancer cell lines, we showed that expression of CCT2 could drive cell proliferation and invasiveness, overcoming the initial slowing of growth caused by the lentiviral transduction system^41^. Most of the overexpressed CCT2 was incorporated in the chaperonin oligomeric complex and variably influenced the levels of other CCT subunits. Depleting CCT2 variably decreased other CCT subunits, and cell viability was reduced as a consequence. Inducible loss of CCT2 in tumor cells implanted in mice impaired tumor growth, indicating that CCT2 is essential for the *in vivo* replication of tumor cells. CCT is thus a viable target for therapeutic intervention in cancer due to its function as a critical protein-folding complex, and the inhibition of CCT could be achieved through direct targeting of the CCT2 subunit.

The function of CCT in support of the cytoskeleton is well known but its activity promoting other cell functions is still being determined. Actin and tubulin are obligate client proteins and loss of CCT activity is known to change cell motility, morphology, and proliferation^42^. We found that overexpressing CCT2 promoted the migration of cells that were not inherently motile, which is in line with our previous report of loss of actin and tubulin upon targeting of CCT with CT20p^16,43^. The use of siRNAs to target different CCT subunits was previously explored by others in select cell lines and resulted in growth arrest that was independent of checkpoint inhibition and reductions in tubulin but not actin^42^. Further evidence supporting the important role of CCT in cell cycling come from studies showing that key cell cycle mediators like Cdc20^44^, CDK2/cyclin E^45^, Polo-like kinase 1 (PLK1)^46^ and others are folded and assembled by CCT^30,33^, which in part explains why CCT levels increase during S phase^47^. Moreover, the CCT chaperonin was recently found to have role in the disassembly of mitotic checkpoint complexes^48^ as well as mediating calcium signaling through Orai1 trafficking^49^. Our finding that overexpressing CCT2 promoted the proliferation of breast cancer and breast epithelial cells is among the first to demonstrate that an increase in cancer cell growth and upregulation of select CDKs as well as other CCT subunits resulted from overexpressing this single CCT subunit.

The role of CCT in many diseases, including cancer, is far from fully characterized. Others reported that individual subunits can have monomeric functions, at least in diseased states, which are independent of their role in the CCT complex, making the study of the chaperonin’s function, regulation, and activity challenging. One study that examined CCT levels in different cell lines found that cancer cells generally had higher expression of CCT protein, but this did not always correlate with protein-folding activity^50^. Another group found that overexpressing CCT1 in yeast did not affect levels of assembled complex, but that the CCT1 subunits remained soluble in the cytosol and had inherent protein-folding activity^51^. While the oligomeric activity of CCT is the most investigated, the ability of select CCT subunits to act as monomers is supported by several studies. For example, CCT4 produced a protusion phenotype by interactions with p150glued and microtubules^52,53^. CCT5 and CCT8 colocalized with actin fibers outside of the oligomer^54^, and CCT5 played a role in the transcriptional regulation of actin^55^. However, the monomeric activity of CCT2 is unknown. Our study revealed that the overexpressed FLAG-tagged CCT2 subunit associated with other CCT subunits, promoting functional effects such as migration and proliferation that are suggestive of increased protein-folding activity. Although this is indicative of oligomeric-driven effects, this does not rule out possible CCT2-driven monomeric activities, such as interactions with the actin filament capping and severing protein, gelsolin^56^. While we found that most of the CCT2-FLAG in cells was in the oligomeric complex, some also was in monomeric form, and could be further studied. Additionally, we showed that silencing of *cct2* gene expression reduced other CCT subunits and increasing CCT2 concomitantly increased CCT subunits. Others found that depletion of CCT subunits (e.g. CCT1, CCT4, CCT5, CCT6) resulted in reduction of the oligomeric complex but not the individual monomers^42,54^. Hence, it remains unclear what regulates CCT subunit expression and chaperonin activity but could involve interplay between other chaperones and partitioning of client proteins^50^ or the response to conditions of stress^57^.

Our research with CCT2 suggests that the individual CCT subunits may have different roles in the activity of the chaperonin complex in cancer cells that remain to be studied. The mechanisms by which CCT subunit expression is regulated are also poorly understood. However, it is clear that CCT is an essential complex needed by cancer cells to survive and grow. Key questions remain – is CCT a general cytosolic chaperone or are its substrates highly restricted, as some studies suggest^30^, and is the activity of the complex driven by the demands for protein folding to support cell cycling, cytoskeletal rearrangements or chromatin remodeling? If the latter, do cancer cells usurp the restricted activities of CCT that are driven by oncogenic processes, becoming more susceptible to inhibition of the chaperonin than healthy cells? Are there unique monomeric activities of CCT subunits? As a step towards answers, our study identifies CCT2 as a potential target for therapeutic inhibition as well as diagnostic development, whose loss could impede the activity of the chaperonin complex in a manner that is detrimental to cancer cells.

## Materials and Methods

### Cell lines and culture conditions

Cell lines used were MDA-MB-231 (ATCC HTB-26) TNBC cells, MCF10A (ATCC CRL-10317™) breast epithelial cells, MCF7 (ATCC HTB-22) ER + breast cancer cells, T47D (ATCC HTB-133) PR + breast cancer, E0771 (CH3 Biosystems) murine TNBC cells, and K562 chronic myelogenous leukemia (CML) cells (ATCC CCL-243). MDA-MB-231 cells were cultured in Dulbecco’s Modified Eagle’s Medium (DMEM) (Corning) supplemented with 10% fetal bovine serum (FBS) (Gemini) and 1% Penicillin-Streptomycin (P/S) (Corning). MCF10A cells were cultured in Mammary Epithelial Basal Medium (MEGM-Lonza) supplemented with bovine pituitary extract (BPE), human epidermal growth factor (hEGF), hydrocortisone (all from MEGM bullet kit - Lonza) and 1% Penicillin-Streptomycin (Corning). MCF7 cells were cultured in Eagle’s Minimum Essential Medium (EMEM) (ATCC) supplemented with 10% FBS (Gemini), 1% P/S (Corning), and 0.01 mg/mL human recombinant insulin (Santa Cruz). T47D cells were cultured in RPMI-1640 (Corning) supplemented with 10% FBS (Gemini), 1% P/S (Corning), and 0.2 units/mL human recombinant insulin (Santa Cruz). E0771 and K562 cells were cultured in RPMI-1640 (Corning) supplemented with 10% FBS (Gemini) and 1% P/S (Corning). Cells were used between passages 2 and 15. The MDA-MB-231 and E0771 cells were transduced with lentiviral particles in the same medium as the wild-type parental cell lines containing tetracycline-free FBS (Takara Biosciences) and 0.5 μg/mL puromycin dihydrochloride (ThermoFisher). The MCF10A, MCF7, T47D, and K562 cells, transduced with the lentiviral particles prepared as described below, were cultured in the same medium as the wild-type parental lines with the addition of 0.5 μg/mL puromycin dihydrochloride (ThermoFisher). All cells were grown in a humidified incubator at 37 °C with 5% carbon dioxide.

### Plasmids

The lentiviral plasmid pLV[Exp]-EGFP:T2A:Puro-EF1A > {hCCT2[ORF023792]*/FLAG for CCT2 expression was purchased from Vector Builder by Cyagen Biosciences. Envelope vector pMD2.G (Addgene 12259) and packaging vector psPAX2 (Addgene 12260) were provided by Didier Trono through Addgene plasmid repository. Control plasmid for expression of pLV[Exp]-EGFP:T2A:Puro-EF1A> was also purchased from Vector Builder and provided pre-packaged. The lentiviral plasmid for inducible depletion of CCT2 targets the TTAGAATAGTGGCACCATC mature antisense and is SMARTVector Inducible shRNA (Dharmacon). Promoter used was mCMV and expressed TurboGFP. Control plasmid (non-targeting) also expresses TurboGFP with the mCMV promoter. Lentiviral particle preparations were purchased (Dharmacon). CCT2 depletion and GFP expression were induced by doxycycline at a dose of 0.5 µg/mL.

### Lentiviral transduction

To generate viral particles for CCT2-FLAG expression, lentiviral plasmid pLV[Exp]-EGFP:T2A:Puro-EF1A > {hCCT2[ORF023792]*/FL (Cyagen Biosciences), envelope vector pMD2.G (Addgene 12259), and packaging psPAX2 (Addgene 12260) were transfected into 293 T cells using transfection solution (105 μM Tris, water, 98 mM CaCl2, and 1X HEPEs-buffered saline). Supernatants containing the virus were harvested at 24 and 48 hours post-transfection. The virus was concentrated using 15 mL Beckman Ultraclear tubes and centrifuged at 4 °C 70000 x g for 1.5 hours followed by resuspension in RPMI 1640 (Corning). To generate CCT2-FLAG expressing cells, MCF10A, MCF7, and T47D cells were plated in 6 well plates and transduced with 50X virus stock the following day in serum-free media as described above with 8 μg/mL polybrene (Millipore) for 18 hours. The following day, cells were washed twice with 1X phosphate-buffered saline (Corning) and medium replaced with standard media for recovery. On the third day, 1 µg/mL puromycin was added as a selection agent. CCT2-FLAG expression was monitored by tracking the GFP signal in the transduced cells by microscopy and flow cytometry. To inhibit CCT2, MDA-MB-231 and E0771 cells were plated in 6 well plates and transduced with viral particles at MOI: 0.3 the following day in serum-free media as described above with 8 μg/mL polybrene (Millipore) for 18 hours. The following day cells received additional media with FBS for recovery. On the third day, 1 µg/mL puromycin was used as a selection agent. To induce shRNA expression, 0.5 µg/mL doxycycline was added to the media for 72–96 hours.

### Immunoblots

Lysate preparation, total protein staining, secondary antibodies, blot normalization and relative protein levels calculations were performed as previously described^14^. Modifications from the previously published protocol are noted. Antibodies used: anti-CCTβ (ab109184), anti-TCP-1Δ (ab129072), anti-TCP-1ε (ab129016), and anti-FLAG (ab1162), from Abcam, anti-CCT3 (MA5-27872) from Invitrogen, anti-CCT5 (NBP2-43680) from Novus, anti-TCP-1β (MAB10050) from Millipore, anti CDK2 (78B2) and anti-CDK4 (D9G3E) from CellSignaling, α-tubulin IgG primary antibody (DM1A) from Santa Cruz Biotechnology. Total protein concentration was determined using Pierce BCA Protein Assay Kit (Thermo Scientific #23227) following manufacturer’s protocol for 96 well plate. Lysates were normalized to 1 mg/ml using lysis buffer prior to freezing and ~9–15 μg of total protein was loaded per well. Blot images were captured at the same intensity using the Odyssey Imaging System (LI-COR).

### Migration assay

Cellular migration was measured using Oris Migration Assembly Kit (Platypus Technologies) following manufacturer’s protocol. 30,000 MCF10A cells were plated around the provided plugs in a 96 well plate and allowed to adhere for 5 hours before the plugs were removed and cells cultured for 16 hours. In references wells, plugs were removed directly before staining. For visualization and quantitation of migration, cells were stained with CFSE for 20 min (Thermofisher)^14^ and imaged on the Cytation 5 (Biotek). The images were analyzed using Gen5 Software for mean GFP signal from cells within the area cleared by the plug. The fluorescent signal from each well was quantified by subtracting the pre-migration signal from the post-migration signal.

### Proliferation assay

Cell proliferation was assessed using Biotium ViaFluor® 405 SE Cell Proliferation Kit (Cat #30068-T). MCF10A, MCF7, T47D lentiviral control and CCT2-FLAG cells were plated at 150,000 cells per well in a 6 well plate and incubated overnight. The next day, the cells were stained following manufacturer’s protocol. Cells were incubated for 48 hours and analyzed by flow cytometry (BD Cytoflex). Reference wells were assessed at 0-hour time point. Data analysis was performed using De Novo FCS Express 6 software.

### Cell cycle analysis

Cell cycle analysis was performed using Click-iT™ Plus EdU Flow Cytometry Assay Kit with Alexa Fluor 647 (Thermofisher). 300,000 MCF10A, MCF7, T47D lentiviral control and CCT2-FLAG cells were plated into T-25 tissue culture flask and incubated for 48 hours. 10 µM EdU was added and cells incubated for an additional 6 hours. Cells were collected and processed following manufacturer’s protocol and measured on BD Cytoflex flow cytometer. Analysis was performed using De Novo FCS Express 6 software.

### *In-vivo* experiments

E0771 cells or E0771 CCT2 shRNA cells were orthotopically implanted into the mammary fat pad of female C57Bl/6 mice (n = 4) (Jackson Laboratories) as follows. Each mouse received 250,000 cells in 1:1 PBS and Matrigel (Corning) solution. Four days post-surgery, standard mouse chow was exchanged for 200 mg/kg doxycycline mouse chow (Envigo). Mice were monitored three times a week for tumor development and tumors were measured using calipers. Tumor volume was calculated using the equation volume = height*(width^2)*0.5^58^. At experimental endpoints, mice were humanely euthanized, tumors excised, and placed in 10% neutral-buffered formalin. Tumors were processed, embedded in paraffin wax, and sectioned on Leica equipment. IHC was performed as described below. This experiment was repeated twice. All animal work performed was approved by the University of Central Florida (UCF) Institutional Animal Care and Use Committee (IACUC) under an approved protocol and is in accordance with the National Institutes of Health guidelines for the use of experimental animals. Animals were maintained in conventional housing at the Association for Assessment and Accreditation of Laboratory Animal Care (AALAC)–accredited UCF facility.

### Immunohistochemistry

Murine breast cancer tumors obtained through *in vivo* experiment described above. Samples were stained for CCT2 using anti-CCTβ antibody (LS-B4861; LifeSpan Biosciences) Antibodies were diluted 1:100 in Antibody Diluent (Leica). Staining of tissue arrays was performed using a Bond-Max Immunostainer (Leica), with an epitope retrieval buffer of EDTA pH 9.0 (Leica). Polymer Refine Detection reagents (Leica) were used, which include a hematoxylin counterstain. Scoring of staining was performed by a surgical pathologist as previously published^14,16^.

### Gene expression analysis

The RNA-seq gene expression data of human breast invasive carcinoma patient samples in TCGA were used for correlation analysis with the cancer stage and patient survival time^59,60^. The clinical information of 1110 patients and the 1216 normalized RNA-seq gene expression data (1110 tumor + 116 normal tissue) were downloaded from the Xena Public Data Hubs (https://xena.ucsc.edu). The log2 (x + 1) transformed Transcripts Per Million (TPM) were reported for gene expression levels. Pearson Correlation Coefficients was applied to determine the relation between gene expression level and cancer stage in Fig. 1A and Table 1. The Wilcoxon rank sum test was used to compare the differences between the expression level in breast tissue and triple negative breast cancer tumor samples in Fig. S1. The Kaplan-Meier survival curves were generated based on alterations or gene expression levels in the CCT subunits, and the difference between groups was determined by Log-rank test. The Pearson Correlation Coefficients of the gene expressions between CCT2 and all the other genes across the cancer patients in each cancer stage were calculated. The top-ranked genes that the expression level was highly correlated with CCT2 were listed in Supplemental Table S1. Then the gene set enrichment analysis based on DAVID Bioinformatics Resources^61^ was applied to determine whether the top-ranked genes were over-represented in GO (Gene Ontology) terms, KEGG (Kyoto Encyclopedia of Genes and Genomes) pathways. The enriched functional terms and pathways were also listed in Table S1. These enriched functional profiles and molecular interactions could help us better understand the underlying biological processes related to CCT2 in breast cancer.

### Microscopy

Brightfield images and overlay GFP images were captured on the Biotek Cytation 5. For confocal microscopy imaging, cells were seeded in 6-well culture plates (Corning) containing 25 mm coverslips (Fisher Scientific). Nuclei were stained with 50 μL/coverslip anti-fade DAPI (Invitrogen) and stained with 0.1 µg mouse α-tubulin IgG primary antibody (Santa Cruz Biotechnology and 0.5 μg Alexa Fluor 568-conjugated goat anti-mouse IgG secondary antibody (Invitrogen) per coverslip. Images were acquired with a Zeiss LSM 710 confocal microscope at 100X magnification (Carl Zeiss AG, Oberkochen, GER).

### Viability assay

E0771 cells were seeded in a clear bottom, black walled 96 well plate as follows: 6 wells untreated, 6 wells control shRNA, 6 wells CCT2 shRNA. Once the cells adhered (24 hrs post seeding) 3 wells per group were treated with 0.5 μg/ml doxycycline to induce expression of CCT2 shRNA. Viability was determined 72 hours post induction using Live/Dead (Thermofisher) following manufacturer’s protocol by calculating the ratio of GFP and TexasRed signal. Signal intensity was measured in the Cytation5 instrument (Biotek) and Gen5 software. Signal from 3 wells were averaged as means and standard error^26^.

## Supplementary information

Supplementary Figures.

## Data Availability

Gene expression data used in Fig. 1 was compiled by the Akbani lab^36^, please see reference for availability. The Cancer Genome Atlas (TCGA) data used during the study was obtained via the publicly available https://www.cbioportal.org website.
