# Supplementary material for: Investigating Chaperonin-Containing TCP-1 subunit 2 as an essential component of the chaperonin complex for tumorigenesis
Source: Sci Rep. 2020 Jan 21;10:798. doi: 10.1038/s41598-020-57602-w (PMC6972895; doi:10.1038/s41598-020-57602-w)
Supplement: Supplementary file 1 — Supplementary Figures. [file 41598_2020_57602_MOESM1_ESM.pdf]

Investigating Chaperonin-Containing TCP-1 subunit 2 as an essential component of the chaperonin complex for tumorigenesis

Anne E. Showalter[1#], Ana C. Martini[1#], Daniel Nierenberg[1], Kristen Hosang[1], Naima Ahmed Fahmi[2,3], Priya Gopalan[4], Amr S. Khaled[5], Wei Zhang[2,3], and Annette R. Khaled[1*]

[#]Share equal authorship

[1]Division of Cancer Research, Burnett School of Biomedical Science, College of Medicine, University of Central Florida, Orlando, FL 32827, USA; [2]Department of Computer Science, University of Central Florida, Orlando, FL 32827, USA; [3]Genomics and Bioinformatics Cluster, University of Central Florida, Orlando, FL, 32816, USA; [4]Oncology, Department of Internal Medicine, Orlando VA Medical Center, Orlando, FL 32827, USA; [5]Pathology and Laboratory Medicine, Orlando VA Medical Center, Orlando, FL 32827, USA

[*]Corresponding Author:

Annette R Khaled, PhD

Professor and Head, Division of Cancer Research

Burnett School of Biomedical Sciences

University of Central Florida

6900 Lake Nona Blvd

Orlando, FL. 32827

407-266-7036

annette.khaled@ucf.edu

Figure S1

A

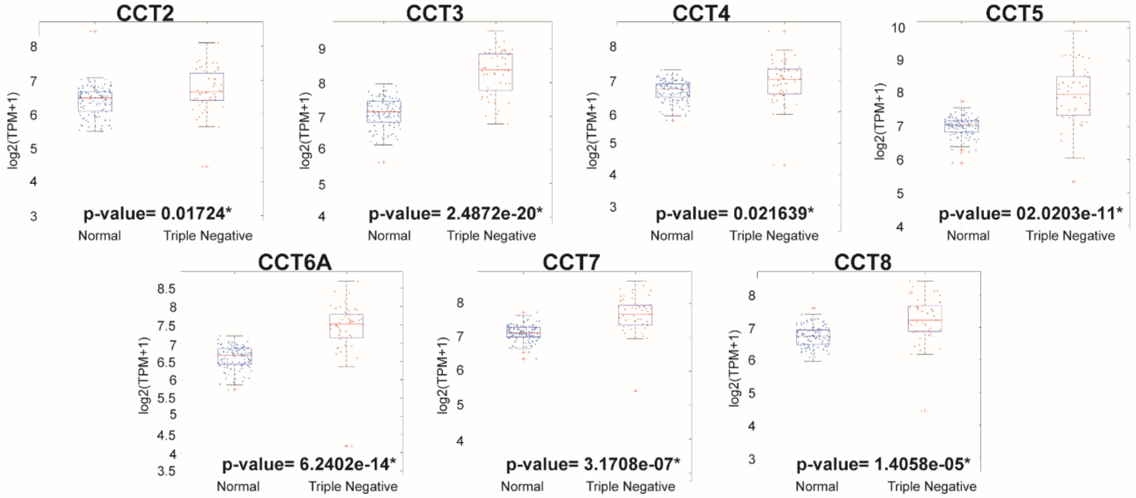

B

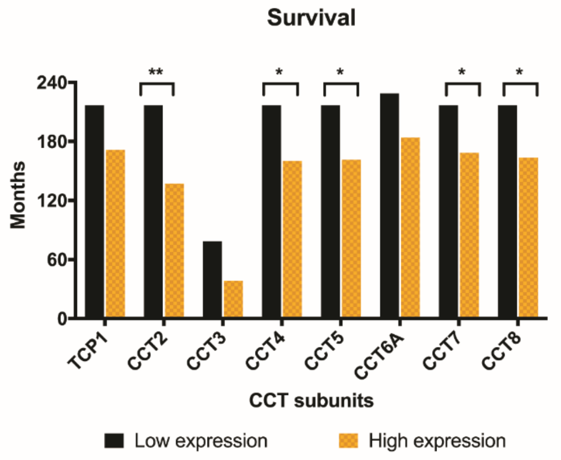

CCT subunits	Low expression cohort (months)	High expression cohort (months)	Significant?	Summary	Adjusted P Value
TCP1	216.7	171.43	No	ns	0.0594
CCT2	216.7	137	Yes	**	0.0026
CCT3	78.48	38.4	No	ns	0.1032
CCT4	216.7	160.28	Yes	*	0.0194
CCT5	216.7	161.43	Yes	*	0.0216
CCT6A	228.85	184.04	No	ns	0.0624
CCT7	216.7	168.5	Yes	*	0.0438
CCT8	216.7	163.46	Yes	*	0.0264

C

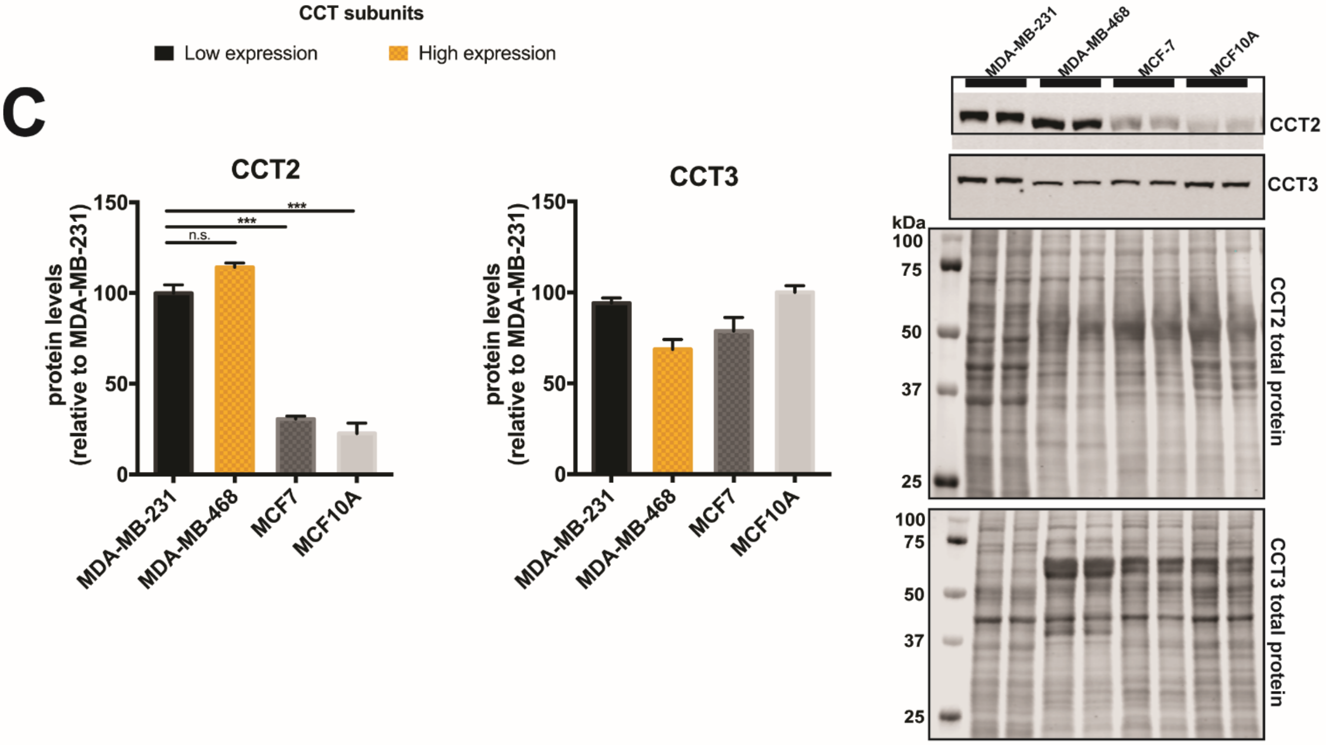

Supplemental Figure S1: High CCT2 levels correlate with decreased patient survival.

A) Plots comparing gene expression levels between triple negative breast cancer (TNBC) tissue and normal tissue for CCT2-8. Expression levels for CCT subunits 2-8 are significantly higher in TNBC tissue than normal tissue. B) Bar graph showing survival data in breast cancer patients with high and low expression of CCT subunits. CCT2 levels had the most significant impact on survival compared to the other 7 subunits. The p-values and analysis summary are also shown. Data obtained using the KM plotter database. C) Relative levels of CCT2 and CCT3 in MDA-MB-231, MDA-MB-468, MCF7 and MCF10A, were determined by western blot (cropped blot is shown). CCT2 levels varied significantly amongst the cell lines while the changes in CCT3 levels were not as significant. Band signal was corrected using total protein and normalized to MDA-MD-231 levels. The graphs show the mean (with s.e.m.) of one representative experiment in duplicates. Analysis performed with Prism GraphPad, using 2-way ANOVA and Dunnett's multiple comparison test. MDA-MB-231 vs MCF7 p-value=0.006 and MDA-MD-231 vs MCF7 p-value= 0.0004. D) Full blots corresponding to cropped images shown in (C). Box shows area of the blot that was cropped.

Figure S2

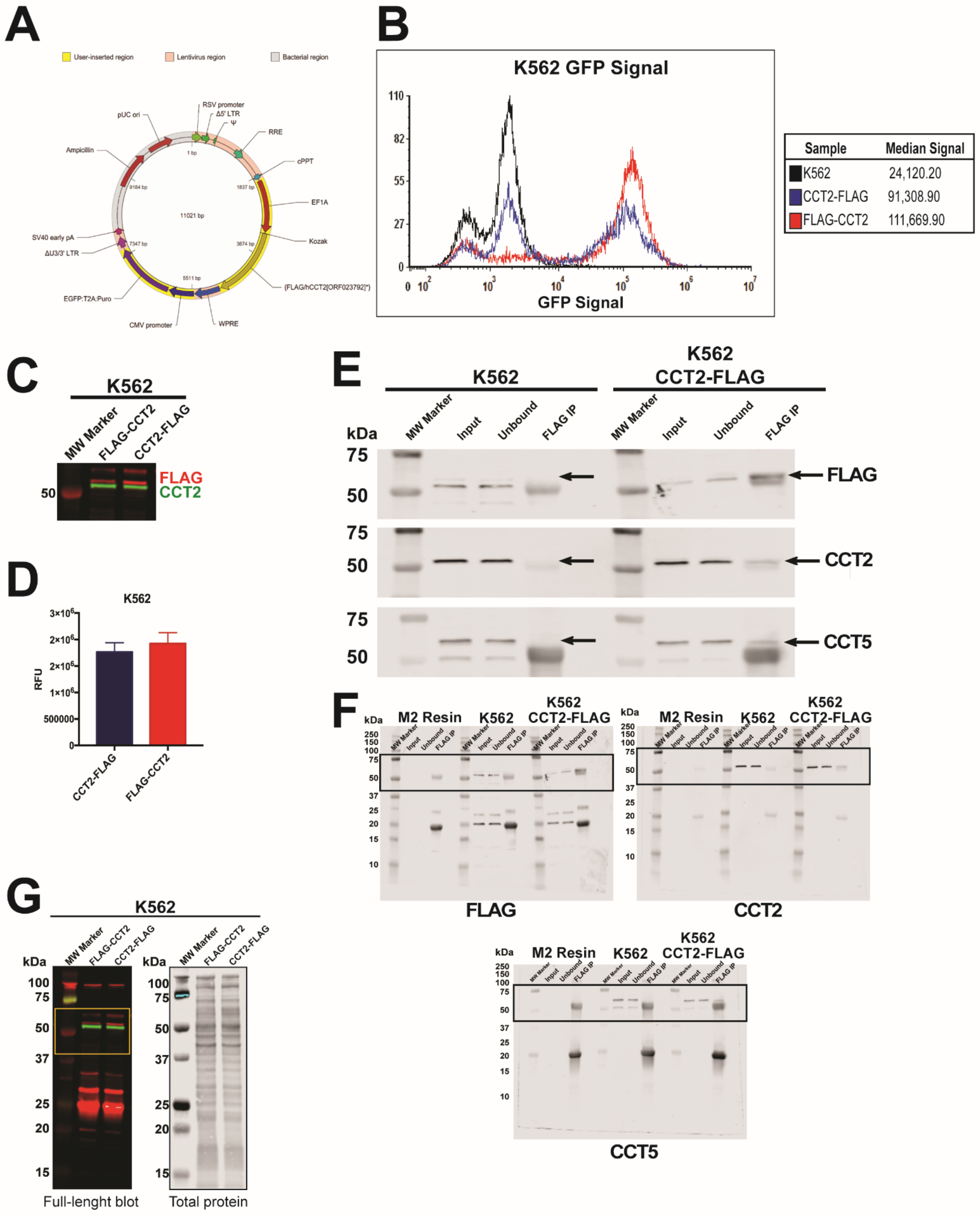
A
User-inserted region
Lentivirus region
Bacterial region
pUC ori
RSV promoter
Δ5' LTR
Ψ
RRE
cPPT
Ampicillin
EF1A
Kozak
SV40 early pA
ΔU3/3' LTR
EGFP:T2A:Puro
CMV promoter
WPRE
{FLAG/hCCT2[ORF023792]*}
11021 bp
B
K562 GFP Signal
GFP Signal
Sample
Median Signal
K562 24,120.20
CCT2-FLAG 91,308.90
FLAG-CCT2 111,669.90
C
K562
MW Marker
FLAG-CCT2
CCT2-FLAG
FLAG
CCT2
D
K562
RFU
CCT2-FLAG
FLAG-CCT2
E
K562
K562 CCT2-FLAG
MW Marker
Input
Unbound
FLAG IP
kDa
FLAG
CCT2
CCT5
F
M2 Resin
K562
K562 CCT2-FLAG
FLAG
CCT2
CCT5
G
K562
MW Marker
FLAG-CCT2
CCT2-FLAG
kDa
Full-lenght blot
Total protein

Supplemental Figure S2: CCT2 overexpression using a lentiviral system

A) Two versions of the plasmid expressing CCT2 were designed using Vectorbuilder, one in which the FLAG tag flanks the N-terminus of CCT2 and another in which the FLAG tag flanks the C-terminus. B) EGFP was included in the construct to assess transfection efficiency which was equivalently high in both versions as determined by flow cytometry. C) K562, K562-FLAG-CCT2 and K562-CCT2-FLAG cell lysates were blotted for FLAG and CCT2 to confirm the tagged version of CCT2 protein was being made. Note that endogenous CCT2 is also present. D) Signal from FLAG western blot was normalized to total protein. Both constructs yielded similar levels of FLAG tagged CCT2 as shown by the bar graph. The graph shows the mean (with s.e.m.) of one representative experiment. E) M2 FLAG Resin was used to pulldown FLAG tagged CCT2 in K562-CCT2-FLAG cells and pulldowns were immunoblotted for FLAG, CCT2 and CCT5. Input lanes indicate the original whole cell protein lysate used. Unbound lanes show supernatant after binding with anti-FLAG agarose. FLAG IP indicate the pulldown. Arrows indicate specific bands for FLAG, CCT2, CCT5 while non-specific band below is found in K562 pulldown as well as a product of the M2 agarose. Blots were cropped for clarity to show the relevant bands. F) The complete blots for the cropped blots in E are shown. Box indicates the area cropped for (E). All blots were captured at the same intensity. G) Image of the complete blot corresponding to the cropped blot shown in C. Box indicates the area cropped. The red bands correspond to FLAG antibody which contains high background (non-specific biding). The green bands correspond to CCT2. Blot of the total protein used for normalization is shown.

Figure S3

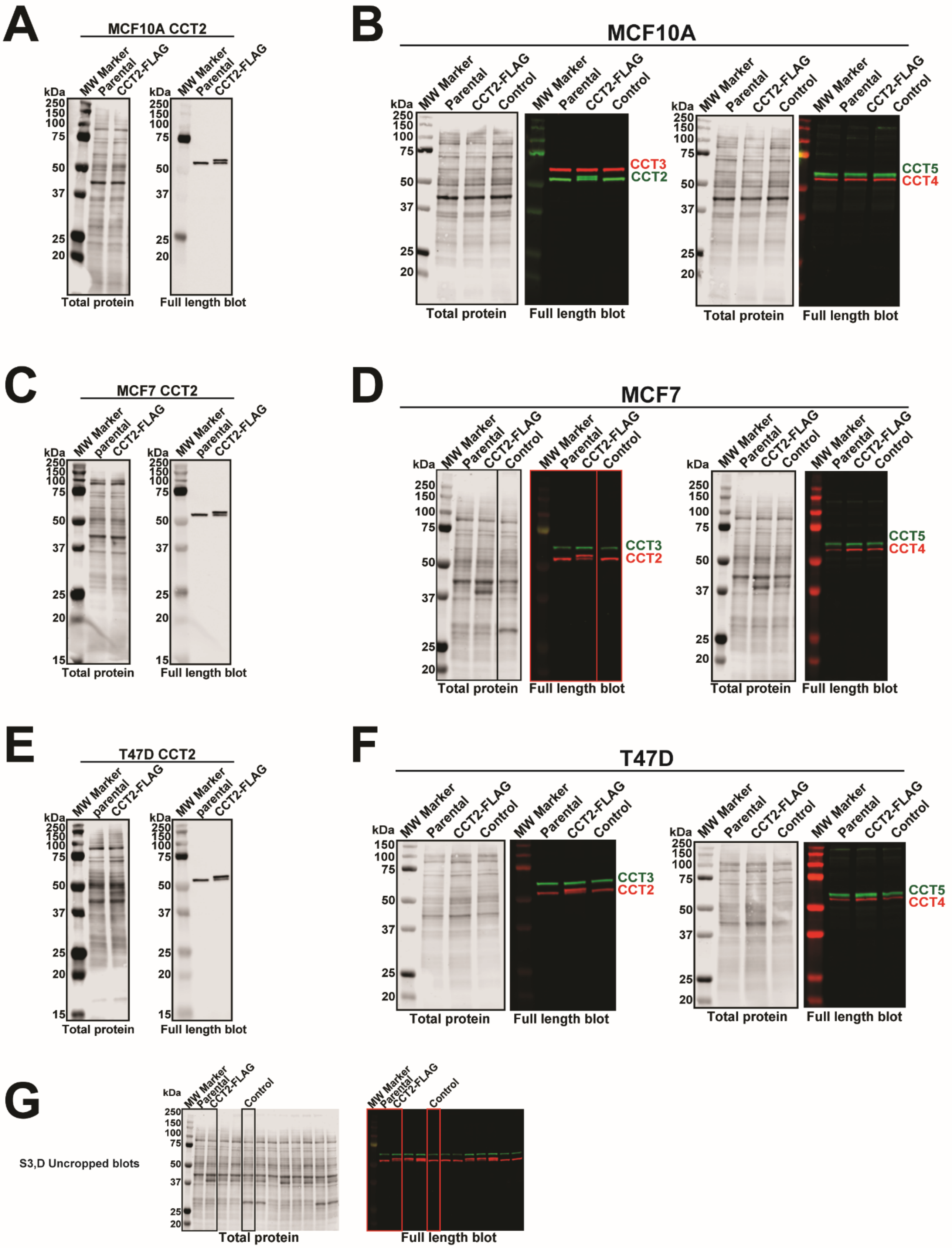
A
MCF10A CCT2
MW Marker
Parental
CCT2-FLAG
kDa
250
150
100
75
50
37
25
20
Total protein
Full length blot
B
MCF10A
MW Marker
Parental
CCT2-FLAG
Control
CCT3
CCT2
CCT5
CCT4
Total protein
Full length blot
C
MCF7 CCT2
MW Marker
parental
CCT2-FLAG
15
Total protein
Full length blot
D
MCF7
MW Marker
Parental
CCT2-FLAG
Control
CCT3
CCT2
CCT5
CCT4
Total protein
Full length blot
E
T47D CCT2
MW Marker
parental
CCT2-FLAG
Total protein
Full length blot
F
T47D
MW Marker
Parental
CCT2-FLAG
Control
CCT3
CCT2
CCT5
CCT4
Total protein
Full length blot
G
S3,D Uncropped blots
MW Marker
Parental
CCT2-FLAG
Control
Total protein
Full length blot

Supplemental Figure S3: Images of representative western blots used for the analysis shown in Figure 3

A, C, E) Total protein stain as well as full blot image corresponding to the blots shown in Figure 3A, 3C and 3E respectively. Parental = not-transfected. B, D, F) Western blots for CCT2, CCT3, CCT4 and CCT5 and the corresponding total protein stain for MCF10A, MCF7 and T47D used for normalization. Note that for panel D, blots were cropped, and lanes of interest pasted next to each other. Cropped lines are shown in black for total protein and in red for CCT2/CCT3 blot. G) Entire blots corresponding to panel D showing where the lanes were cropped.

Figure S4

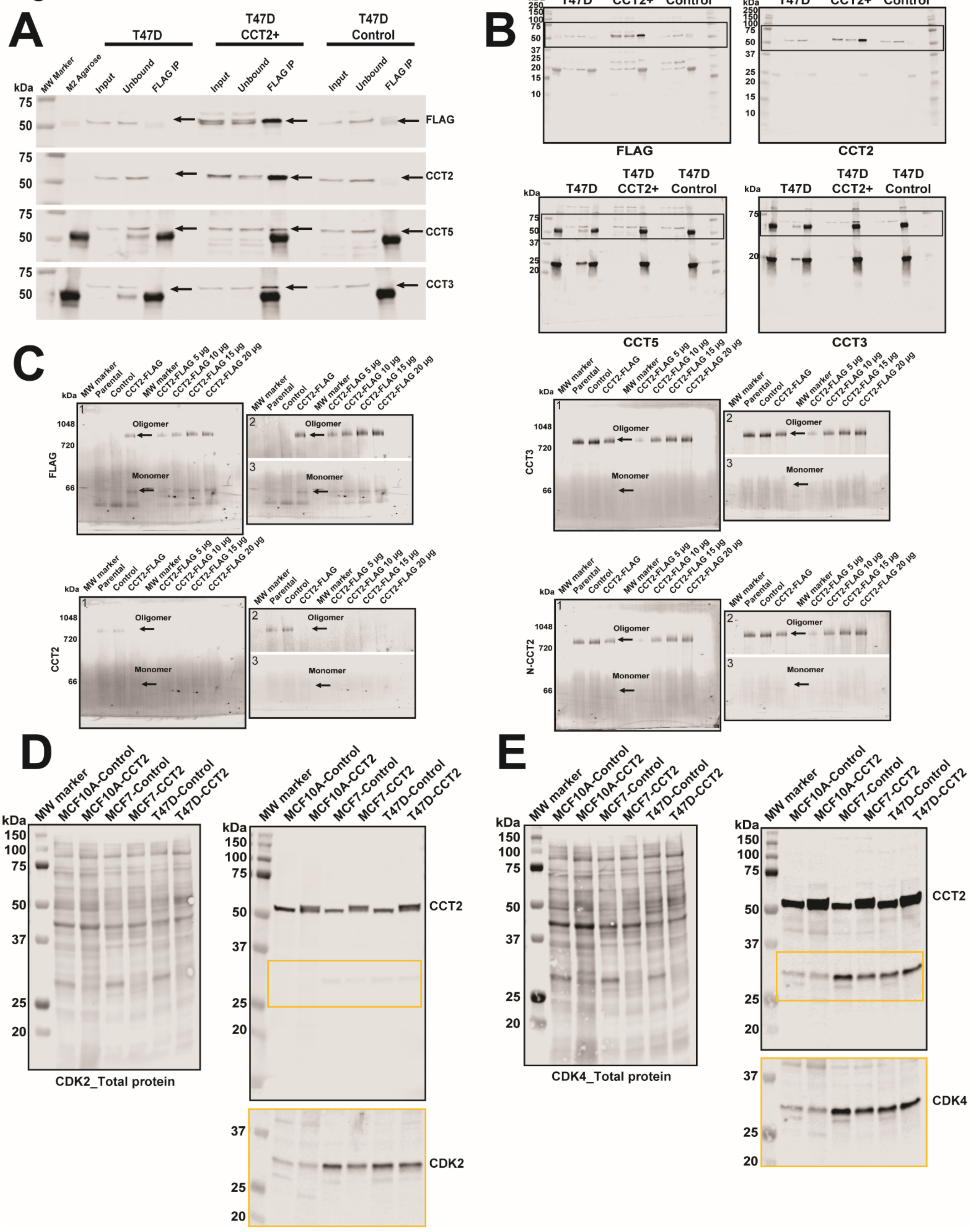

A
T47D
T47D CCT2+
T47D Control
kDa
MW Marker
M2 Agarose
Input
Unbound
FLAG IP
75
50
FLAG
CCT2
CCT5
CCT3
B
T47D
T47D CCT2+
T47D Control
kDa
250
150
100
75
50
37
25
20
15
10
FLAG
CCT2
CCT5
CCT3
C
MW marker
Parental
Control
CCT2-FLAG
CCT2-FLAG 5 µg
CCT2-FLAG 10 µg
CCT2-FLAG 15 µg
CCT2-FLAG 20 µg
1048
720
66
Oligomer
Monomer
FLAG
CCT2
CCT3
N-CCT2
D
MW marker
MCF10A-Control
MCF10A-CCT2
MCF7-Control
MCF7-CCT2
T47D-Control
T47D-CCT2
CDK2_Total protein
CCT2
CDK2
E
CDK4_Total protein
CCT2
CDK4

Supplemental Figure S4: T47D CCT2-FLAG pulldown, T47D native gel, and CDK2/CDK4 immunoblots.

Immunoprecipitation with anti-FLAG M2 agarose using T47D, T47D CCT2-FLAG, and T47D lentiviral control cells is shown above. A) Input lanes indicate the original whole cell protein lysate used. Unbound lanes show supernatant after binding with anti-FLAG agarose. FLAG immunoprecipitate (IP) indicate the pulldown. IP was blotted for FLAG, CCT2, CCT5, and CCT3. Arrows indicate the unique bands found in the T47D CCT2-FLAG IP lane while the non-specific bands can be seen in all IP lanes. CCT3 and CCT5 were found in the CCT2-FLAG pulldown lanes indicating incorporation into the CCT complex. Blots were cropped for clarity to show the relevant bands. B) The complete blots for the cropped blots in A are shown. Blots were captured at the same intensity. C) Native gel using T47D, T47D CCT2-FLAG, and T47D lentiviral control cells shows the presence of CCT2-FLAG, measured by FLAG, in both the CCT oligomeric complex and as a monomer. Measurements comparing the total FLAG to monomeric FLAG found approximately 24% exists in monomer form. Endogenous CCT2 (CCT2), CCT3, and total CCT2 (N-CCT2) are shown to be in the oligomer but not existing in a monomeric form. The N-CCT2 antibody may not bind to its epitope in the monomer native conformation. Each blot shows 1) The entire blot, 2) the CCT oligomer at ~900 kDa, and 3) the monomeric subunit at ~60 kDA. All images were taken at the same intensity but instrument adjustments allow the bands to be more visible. The staining of the lanes at the bottom of the image is G-250 from running the gel that was not removed even after destaining. D-E) Total protein blots as well as full blot corresponding to CDK2 and CDK4 cropped blots in Figure 4 are shown. CCT2 was also detected in the same blots, as CDK2 (or CDK4) using the same secondary antibody for visualization. Note that CCT2 is more abundant than the CDK targets and the Odyssey software automatically adjusts for the brightest pixel, hence the bottom half of the blots for the CDKs were imaged individually. Full blot and cropped blots are shown.

Figure S5

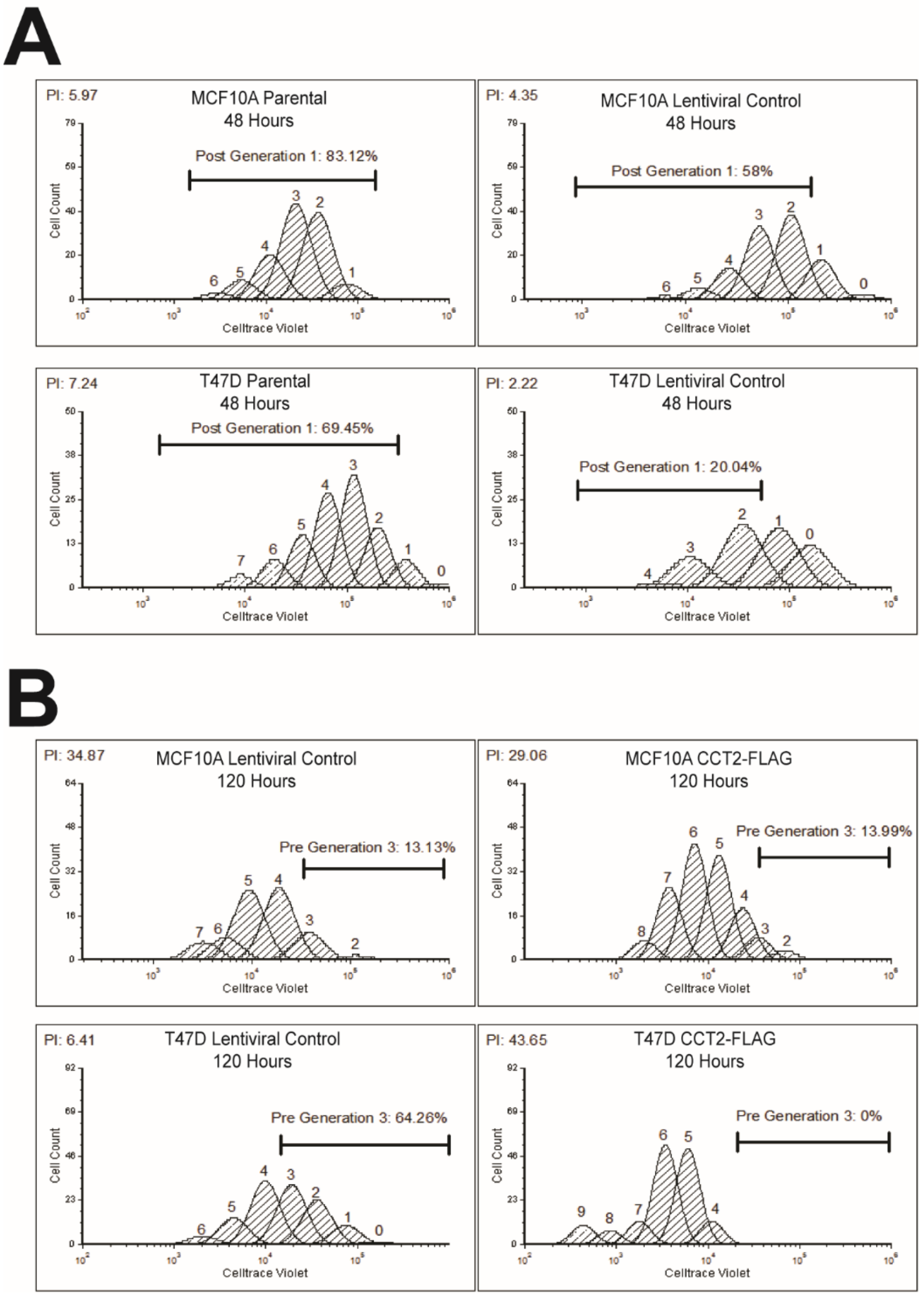

Supplemental Figure S5: Proliferation of parental cells is higher than lentiviral control cells at 48 hours, while CCT2-FLAG cells proliferate more than lentiviral control cells at 120 hours.

A) MCF-10A (parental) and MCF-10A lentiviral control cells were assessed for proliferation by labeling with CellTrace Violet for 48 hours using flow cytometry (CytoFlex) and generation time determined using FSC Express 6 software. B) MCF-10A CCT2-FLAG, T47D CCT2-FLAG and control lentiviral cells were assessed for proliferation as in (A) but cells were labeled with CellTrace Violet for 120 hours.

Figure S6

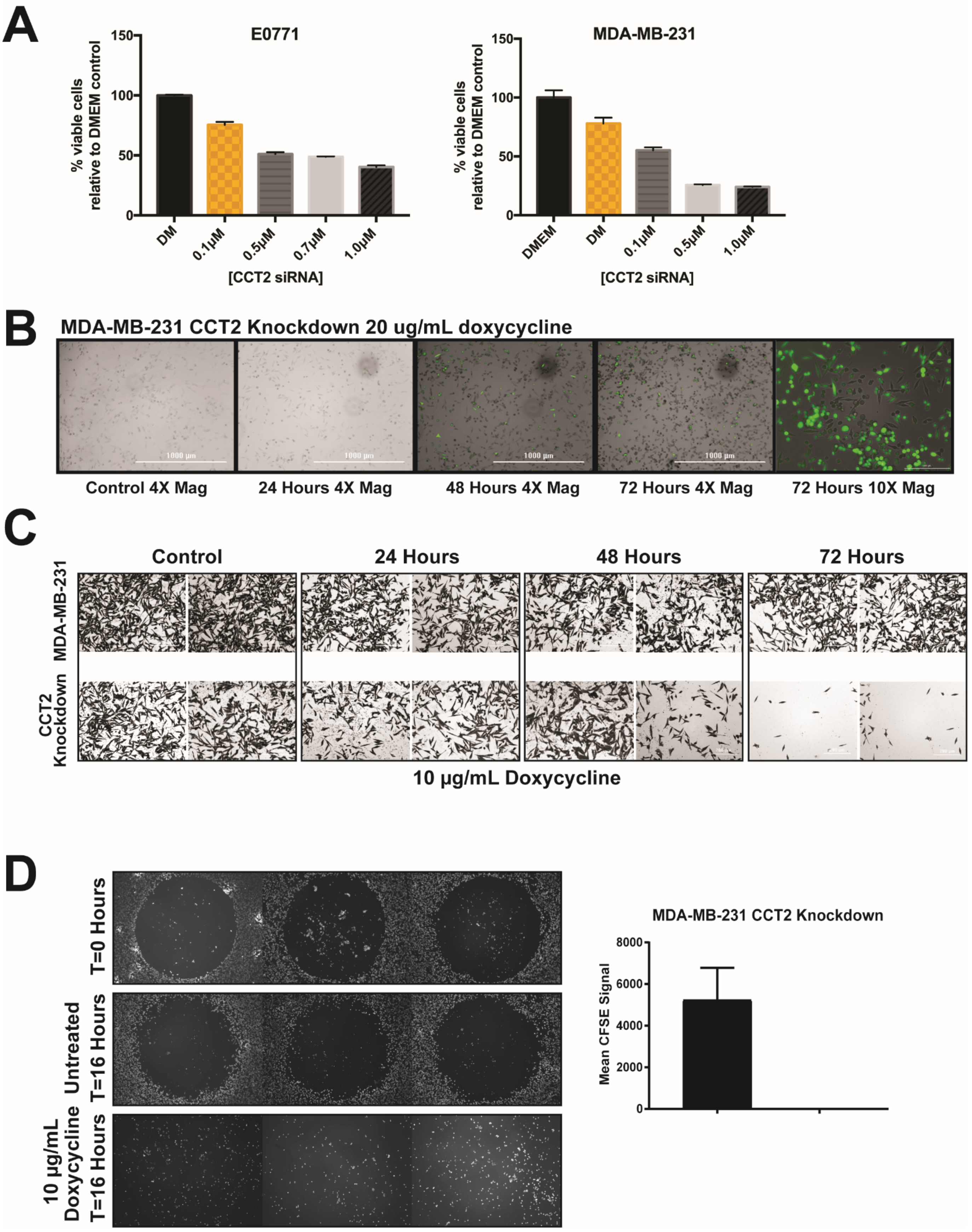
A
E0771
% viable cells relative to DMEM control
150
100
50
0
DM
0.1μM
0.5μM
0.7μM
1.0μM
[CCT2 siRNA]
MDA-MB-231
DMEM
B
MDA-MB-231 CCT2 Knockdown 20 ug/mL doxycycline
1000 μm
Control 4X Mag
24 Hours 4X Mag
48 Hours 4X Mag
72 Hours 4X Mag
72 Hours 10X Mag
C
Control
24 Hours
48 Hours
72 Hours
MDA-MB-231
CCT2 Knockdown
10 μg/mL Doxycycline
D
T=0 Hours
Untreated T=16 Hours
10 μg/mL Doxycycline T=16 Hours
MDA-MB-231 CCT2 Knockdown
Mean CFSE Signal
8000
6000
4000
2000

Supplemental Figure S6: Knocking down CCT2 leads to cell death

A) Bar graph showing that CCT2 knockdown using siRNA leads to cell death in a dose dependent manner. MDA-MB-231 and E0771 cells were subjected to increasing concentrations of CCT2 siRNA and cell viability was measured after 72 hours of siRNA treatment using Live-Dead stain as described in methods. B) A doxycycline inducible lentiviral system was then used for controlled knockdown of CCT2 in MDA-MB-231 cells. Transduced cells were imaged 24, 48 and 72 hours post induction. The GFP signal increased overtime indicating expression of CCT2 shRNA. Based on images and confluency, 72 hours post-induction was determined as optimal for subsequent experiments. Magnification used: 10X and 4X. C-D) Adherence of cells from (B) were assessed by crystal violet assay (2 representative images from areas of the well) (C) and migration (D) by Oris migration assay as described in the Methods section. The doxycycline treated cells after 16 hours are detached from the plate.

Figure S7

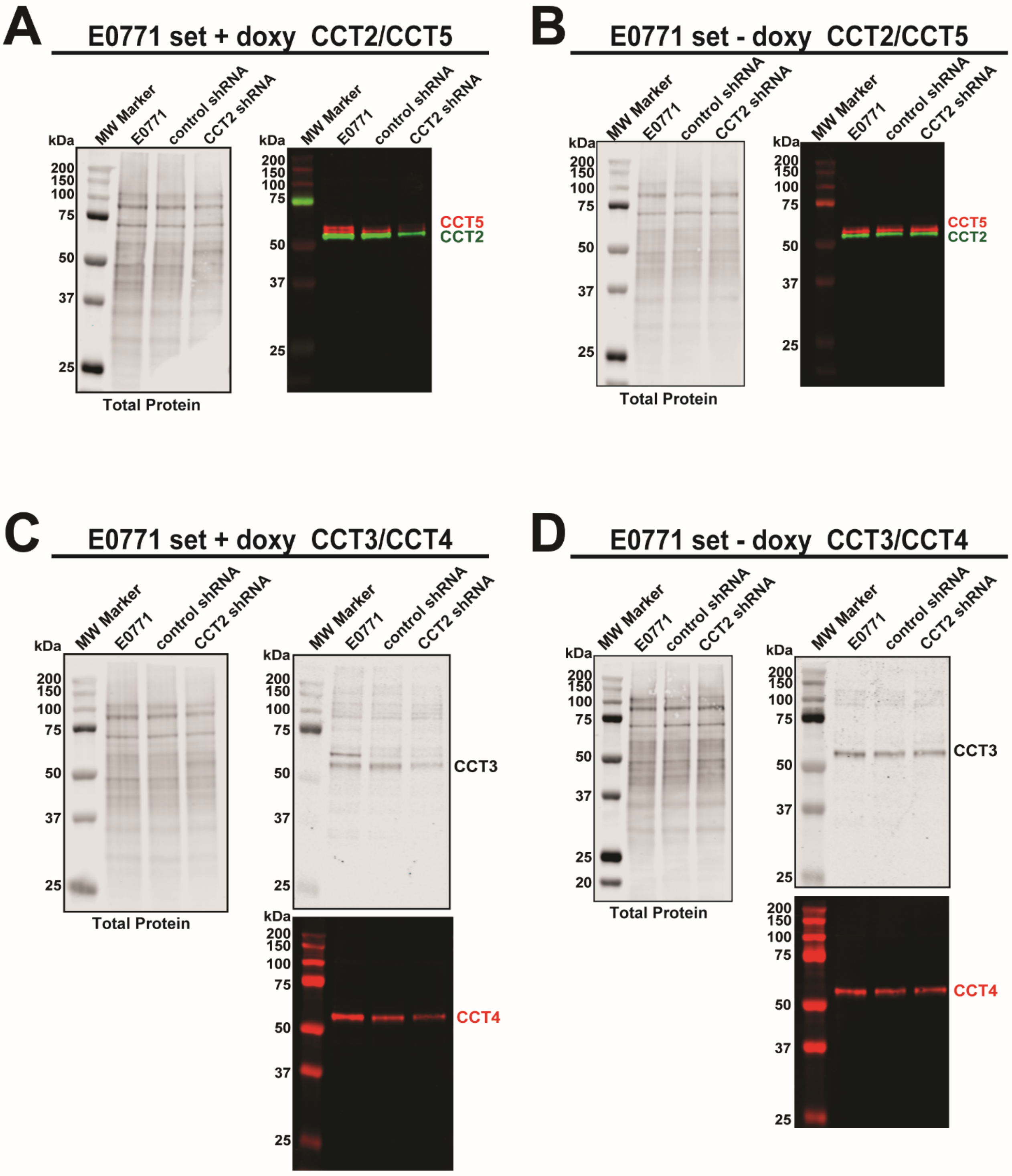

A
E0771 set + doxy CCT2/CCT5
MW Marker
E0771
control shRNA
CCT2 shRNA
kDa
200
150
100
75
50
37
25
Total Protein
CCT5
CCT2
B
E0771 set - doxy CCT2/CCT5
C
E0771 set + doxy CCT3/CCT4
CCT3
CCT4
D
E0771 set - doxy CCT3/CCT4
20

Supplemental Figure S7: Images of representative western blots used for the analysis shown in Figure 6.

E0771 (non-transduced), E0771 control shRNA and E0771 CCT2 shRNA either treated with 0.5µg/ml doxycycline (+doxy) or not (-doxy) were collected and lysates made. ~9µg of total protein was loaded per well, in duplicates. Total protein was used for normalization of band signal. A) Band signal for CCT2 shRNA is less intense than in E0771 and control, indicating CCT2 knockdown. CCT5 band signal was also less intense. B) In the absence of doxy, levels of CCT2 and CCT5 remain constant. C) Signal for CCT4 and CCT3 decreased when CCT2 is silenced. D) In the absence of doxy, CCT3 and CCT4 protein levels remain constant. Full blots are shown from which cropped images were made for Figure 5.

Supplemental Methods

Data Mining and Statistical Analysis

Kaplan-Meier Plotter (KMPlot) repository available http://kmplot.com/analysis/index.php?p=service&cancer=breast

Immunoblots

Performed as described in main paper with the following difference: anti-CCT3 (Sigma HPA006543) was used for blots shown in Supplemental Figure S1.

Proliferation Assay

Performed as described in main paper on MCF10A and T47D parental, lentiviral control, and CCT2-FLAG cells.

FLAG Immunoprecipitation

K562, K562 CCT2-FLAG, T47D, T47D CCT2-FLAG, and T47D lentiviral control cells were collected in equal amounts and lysed to collect protein content. The protein lysate was incubated with pre-equilibriated M2 anti-FLAG agarose resin for 1 hour at room temperature while spinning. The unbound protein lysate supernatant is collected and the resin washed three times in 1X HEPES-buffered saline (HBS) (20 mM HEPES pH 7.5, 150 mM sodium chloride, 1 mM EDTA). Resin is boiled in 2X loading buffer (Licor) with 10% β-mercaptoethanol to release proteins and proteins are immunoblotted following the procedure above.

siRNA Transduction

A set of 4 siRNA targeting different regions of CCT2 transcript were pooled together (A-020107-14- 0005 ,A-020107-15 -0005, A-020107-16 -0005 , A-020107-17-0005) , siRNA buffer and Accel Delivery Media (Cat #B-005000) were purchased from Dharmacon. Followed the protocol as described by the manufacturer. MDA-MB-231 or E0771 cells were seeded at 5000 cells/well in a 96 well clear bottom black walled plate and incubated overnight in 100 µLs of their growth medium, at 37ºC, 5% CO_2. The following day growth medium was replaced by ADM containing the indicated concentrations of CCT2 siRNA, ADM only or growth medium only in triplicates. Plates were incubated for 72 hours. Viability was determined by Live/Dead (Thermofisher) as described in the main paper.

Adhesion assay

MDA-MB-231 cells were plated in tissue-culture treated clear 96 well plates (Eppendorf) and incubated as described above. For the assay, plates were shaken at 1400 rpm on a plate shaker for 15 seconds to remove any partly attached cells. Media was aspirated off and wells washed with 0.1% bovine serum albumin (BSA) in DMEM two times. Cells were fixed using 10% neutral-buffered formalin (Leica) for 15 minutes and washed with 0.1% BSA in DMEM. Cells were stained using 5 mg/mL crystal violet stain (Fisher) for 10 min and rinsed with distilled water. Once the plates dried, absorbance was read at 595 nm

on Cytation 5 plate reader (Biotek). Statistical significance was determined using unpaired student's T-test and was defined as $p < 0.05$.

Migration Assay

Performed as described in the main paper using MDA-MB-231 cells.

Native PAGE

Cell pellets were collected and lysed in a solution of 5% digitonin (Sigma) and 1X NativePAGE™ (Thermo) Sample buffer for ten minutes on ice. Lysates were spun at 16000 x g for 30 minutes at 4° C to clear debris. Protein concentration calculated as described above. To run native gel, samples were prepared with 1:5 NativePAGE™ 5% G-250 additive and loaded onto NativePAGE™ Novex® 3-12% Bis-Tris gel. Gel was run at 150V using NativePAGE™ Anode and Cathode buffers following provided protocol until dye front reached the bottom. Wet transfer using NuPAGE® transfer buffer (Thermo) for one hour at 25V transferred proteins to low-fluorescence PVDF membrane (Millipore). The membrane was destained in 8% acetic acid for fifteen minutes at room temperature, air dried, and reactivated in methanol. Immunoblot performed as described.
